# Neuroimaging Signatures of Temporomandibular Disorder and Burning Mouth Syndrome: A Systematic Review

**DOI:** 10.3390/dj13080340

**Published:** 2025-07-24

**Authors:** Sarah Fischer, Charalampos Tsoumpas, Pavneet Chana, Richard G. Feltbower, Vishal R. Aggarwal

**Affiliations:** 1 School of Dentistry, Faculty of Medicine & Health, University of Leeds, Worsley Building, Clarendon Way, Leeds LS2 9LU, UK; s.e.fischer@leeds.ac.uk (S.F.); pavneet.chana@nhs.net (P.C.); 2 Department of Nuclear Medicine and Molecular Imaging, University Medical Center Groningen, University of Groningen, 9713 GZ Groningen, The Netherlands; c.tsoumpas@umcg.nl; 3 Child Health Outcomes Research at Leeds (CHORAL), Leeds Institute for Data Analytics, School of Medicine, University of Leeds, Leeds LS2 9LU, UK; r.g.feltbower@leeds.ac.uk

**Keywords:** chronic orofacial pain, neuroimaging, temporomandibular disorder, burning mouth syndrome, ICD-11 classification, central sensitisation

## Abstract

**Background:** Chronic primary orofacial pain (COFP) affects approximately 7% of the population and often leads to reduced quality of life. Patients frequently undergo multiple assessments and treatments across healthcare disciplines, often without a definitive diagnosis. The 2019 ICD-11 classification of chronic primary pain clusters together COFP subtypes based on chronicity and associated functional and emotional impairment. **Objective:** This study aimed to evaluate whether these subtypes of COFP share common underlying mechanisms by comparing neuroimaging findings. **Methods:** A systematic review was conducted in accordance with PRISMA guidelines. Searches were performed using Medline (OVID) and Scopus up to April 2025. Inclusion criteria focused on MRI-based neuroimaging studies of participants diagnosed with COFP subtypes. Data extraction included participant demographics, imaging modality, brain regions affected, and pain assessment tools. Quality assessment used a modified Coleman methodological score. **Results:** Fourteen studies met the inclusion criteria, all utilising MRI and including two COFP subtypes (temporomandibular disorder and burning mouth syndrome). Resting- and task-state imaging revealed overlapping alterations in several brain regions, including the thalamus, somatosensory cortices (S1, S2), cingulate cortex, insula, prefrontal cortex, basal ganglia, medial temporal lobe, and primary motor area. These changes were consistent across both TMD and BMS populations. **Conclusions:** The findings suggest that chronic primary orofacial pain conditions (TMD and BMS) may share common central neuroplastic changes, supporting the hypothesis of a unified pathophysiological mechanism. This has implications for improving diagnosis and treatment strategies, potentially leading to more targeted and effective care for these patients.

## 1. Introduction

Chronic orofacial pain is a common disorder affecting 7% of the population that can lead to poor quality of life, with many patients being unable to perform even simple everyday tasks or attend regular employment [1]. Patients often present to multiple disciplines for help, including primary, secondary, and tertiary care, in both the dental and medical specialities [2,3,4]. Without clear guidelines for diagnosis and management, this often leads to polypharmacy, multiple investigations, and invasive treatments that can cause iatrogenic harm [2,3,4,5,6].

In 2019, Nicholas et al.’s [7] International Classification of Disease (ICD)-11 proposed a new classification of chronic primary pain, which accounts for shared mechanisms based on significant functional and emotional impairment and pain that is chronic (present for 3 months or longer). It recognises the biopsychosocial nature of chronic pain and the significant role psychosocial factors play in perpetuating the vicious cycle of chronic pain, e.g., depression, anxiety, increased frustration, or anger that may interfere with everyday living [7].

Within this classification, chronic temporomandibular disorder (TMD), chronic burning mouth (BMS), and chronic primary orofacial pain (COFP) are recognised as subtypes of chronic primary headache or orofacial pain [7]. Epidemiological evidence [8,9] and the recent International Classification of Orofacial Pain [10] support this classification. That said, there is a dearth of neuroimaging evidence to support this classification of COFP, and it is unclear whether its subtypes share common underlying mechanisms.

The current neuroimaging literature appears to investigate each of these subtypes in isolation, and a previous review focused on overlap between neuralgic pain and TMD [11] rather than COFP subtypes, which are clustered together in the recent ICD-11 classification [7,10]. The aim of the current investigation is therefore to undertake a systematic review of neuroimaging studies of COFP subtypes to determine whether they share common underlying mechanisms.

**Hypothesis:**  *Functional neuroimaging will show that chronic primary orofacial pain subtypes share common underlying mechanisms.*

## 2. Methods

### 2.1. Literature Search and Identification of Studies

**Figure 1:**
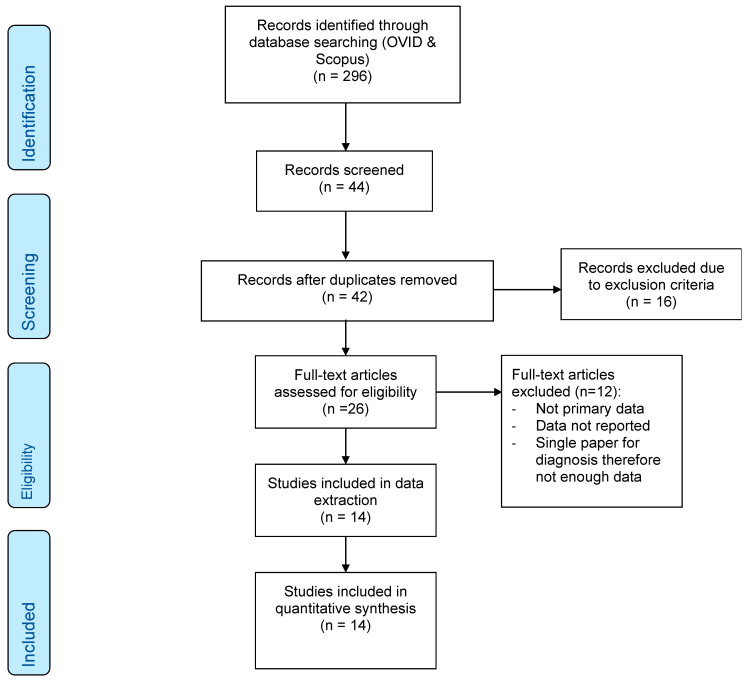
PRISMA flow diagram.

For the literature search, the Preferred Reporting Items for Systematic reviews and Meta-analysis (PRISMA) guidelines were followed [12] (Figure 1). This study was not registered with PROSPERO.

Databases Medline (OVID) and Scopus were used to identify relevant papers published until April 2025. The search strategies for both databases are included in Supplementary File S1. The search strategies focused on chronic primary orofacial pain (COFP) subtypes, namely, chronic temporomandibular disorder (TMD), persistent idiopathic orofacial pain, chronic burning mouth syndrome (BMS), and persistent dentoalveolar pain, as well as previous terms used to describe these conditions, such as atypical facial pain and atypical odontalgia.

### 2.2. Eligibility and Paper Selection

#### 2.2.1. Types of Studies

In the original search, all studies that included relevant neuroimaging of participants with the COFP subtypes described above were included.

#### 2.2.2. Types of Participants

All participants with a confirmed diagnosis of chronic orofacial pain or any of its subtypes, as per the ICD-11 and ICOP, were included [7,10].

#### 2.2.3. Primary Outcome Measure

Primary outcomes included data on structural and functional changes seen on neuroimaging of participants with a diagnosis of chronic orofacial pain. Data measures included functional connectivity, cerebral blood flow, grey matter volume (GMV), fractional anisotropy (FA), brain activation, metabolite levels, peak activation, and white matter structure.

#### 2.2.4. Types of Imaging

Relevant types of neuroimaging included magnetic resonance imaging (MRI), including functional MRI (fMRI), computed X-ray tomography (CT), positron emission tomography (PET), single-photon emission computed tomography (SPECT), and electroencephalogram (EEG).

#### 2.2.5. Types of Stimulation

Both resting-state and task-state neuroimaging results were included. Resting state was defined as the patient not actively doing anything during imaging, either with their eyes open or closed. Task state was defined as any stimulation during imaging and included thermal stimulation, innocuous brushing of the lower lip, finger and thumb, clenching, elicited pain, and Stroop tests.

### 2.3. Data Collection

#### 2.3.1. Selection of Studies

All articles that were identified from the search were screened by two reviewers (SF/PC), and those with irrelevant titles were removed. The abstracts of the remaining articles were read by the reviewers, and any further articles deemed irrelevant were also excluded. Full texts were reviewed, and further exclusions were made at this point.

The following inclusion criteria were used:

- English language, humans, and full text available.
- Neuroimaging of participants definitively diagnosed with a type of COFP.
- Quantitative outcome measures reported.

The following exclusion criteria were used:

- Diagnosis of trigeminal neuralgia, acute orofacial pain, e.g., dental pain, chronic pain.
- Ultrasound or planar X-ray imaging.
- Studies that did not include neuroimaging.
- Chronic pain outside of the orofacial area.
- Review papers.

#### 2.3.2. Data Extraction

A data extraction form created in Microsoft Word enabled systematic extraction of data from each paper manually by two reviewers (SF/PC). Duplicate extraction of a random selection of 10% of the papers was performed by an additional reviewer (VA) to check for accuracy. Any discrepancies were resolved by discussing with a third reviewer (CT). Data extracted included demographics, study eligibility, participant demographics, study inclusion and exclusion criteria, outcomes, and other relevant information.

#### 2.3.3. Data Analysis

Data were analysed by regions of interest that are known to be involved in the pain pathway and the processing of emotions. Relevant brain regions were identified as per the previous literature [11,13] and included the following 10: thalamus (THAL), primary somatosensory cortex (S1), secondary somatosensory cortex (S2), cingulate cortex (CC), insula (INS), prefrontal cortex (PFC), basal ganglia (BG), medial temporal lobe, including hippocampus and amygdala (mTL), primary motor area (M1), and supplementary motor area (SMA).

#### 2.3.4. Assessment of Study Quality

All studies included in this review were assessed and given a score according to the modified Coleman score system [14]. This scoring system generates a score according to study design, methods, and quality of data analysis. It was adapted for this review, and sections that were deemed irrelevant, such as mean follow-up and description of postoperative rehabilitation, were removed, leaving a score out of 100.

## 3. Results

### 3.1. Included Studies/Search Results

Utilising the described search strategy, 296 papers were identified. Subsequent screening was performed based on the paper titles and abstracts, which left a total of 44 papers. Any duplicated papers were removed from this set, which left 42 papers. The exclusion criteria were adhered to, and a further 16 papers were removed, leaving 26 studies for full text assessment. A further 12 studies were excluded because of the following: review articles that did not report their own primary data, participants with a diagnosis other than COFP or its subtypes, articles that did not include neuroimaging, and outcomes of the articles focussing on treatment or management rather than neuroanatomical changes. The remaining 14 articles that met the inclusion criteria were included in this review (Figure 1—PRISMA flow diagram).

### 3.2. Demographics/Study Characteristics

#### 3.2.1. Overview

The patient demographics and study characteristics are all displayed in Table 1. The final 14 papers [15,16,17,18,19,20,21,22,23,24,25,26,27] included two diagnostic subtypes of chronic primary orofacial pain: TMD and BMS. Patients were aged between 18 and 72 years. Eight of the fourteen papers included only female participants, and the remaining six also included male participants. Two of the papers reported the ethnicity of participants [19,20]. The ethnicities included in these papers were Caucasian, Asian, African American, Hispanic, and Arabic.

#### 3.2.2. Pain Duration

The minimum length of time that participants needed to have experienced pain was 3 months. The longest duration of pain reported was 46 years, although five papers did not report the duration of pain.

#### 3.2.3. Confounding

Some of the papers reported other potential confounding factors, such as dominant hand, medications, including the use of hormone replacement therapy (HRT), pain severity, menstrual status, pain outside of the orofacial region, presence of psychological disorders, pain frequency, and site of pain. However, a weakness with all these studies was a failure to adopt a causal inference approach to identify true confounders [28].

#### 3.2.4. Imaging Modalities

All imaging modalities that allow for anatomical and functional imaging were included in the search strategy; however, whilst analysing the papers, it became clear that MRI was the only modality that provided sufficient information to provide a basis for a systematic review. Therefore, the final set of papers is limited to MRI studies.

### 3.3. Risk of Bias Assessment and Quality of Study

Quality assessment of the papers was performed using the modified Coleman methodological score system [14]. Eleven of the papers in the final set scored over 50. Of the remaining papers, Weissman-Fogel et al. [23], Khan et al. [25], and Kohashi et al. [27] scored 44, 49, and 37, respectively.

### 3.4. Assessment of Pain

All the papers included in the final set used a variety of validated pain assessment methods: numerical pain scale, pain catastrophising scale, visual analogue scale, McGill pain questionnaire, symptom checklist 90, brief pain inventory, and state-trait personality inventory.

### 3.5. Resting-State Brain Changes

Resting-state brain changes were imaged using MRI for patients with TMD and BMS. Resting-state instructions for patients varied slightly between studies and included “Close your eyes, stay calm, do not think of anything in particular. Do not fall asleep.” [15], “subjects were instructed to keep their eyes open.” [26], and “subjects were asked to remain awake with their eyes open” [18]. The other papers in this set did not specify resting-state instructions given to patients.

**Figure 2:**
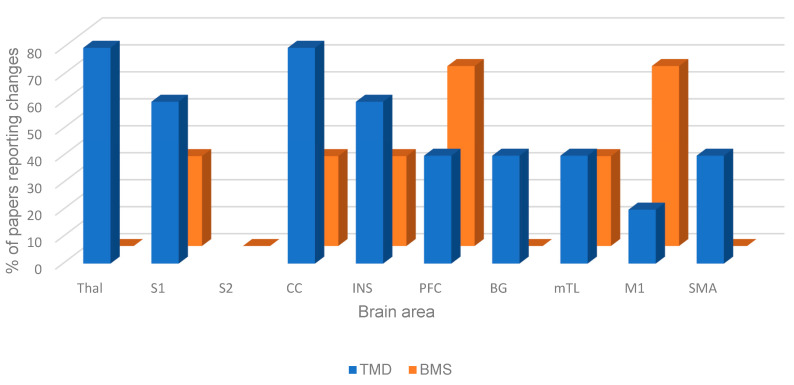
Figure showing resting-state brain changes in TMD and BMS patients.

The most consistent areas reported to have resting-state changes in both TMD and BMS were the primary somatosensory cortex (S1), cingulate cortex (CC), insula (INS), prefrontal cortex (PFC), medial temporal lobe (mTL), and primary motor area (M1). The proportion of papers included in this review that reported resting-state brain changes for both TMD and BMS is shown in Figure 2. The only area of the brain not reported to have resting-state changes in TMD was the secondary somatosensory cortex (S2). For BMS, there were no reported changes in resting state in the thalamus (THA), basal ganglia (BG), and other supplementary areas (SMA).

### 3.6. Task-State Brain Changes

**Figure 3:**
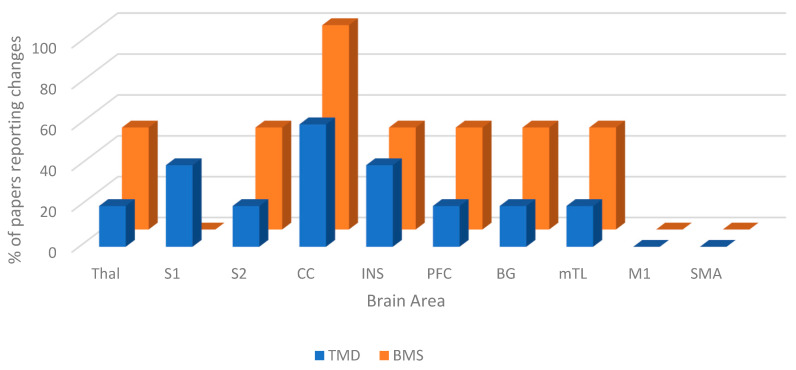
Figure showing task-state brain changes in TMD and BMS.

Task-state brain changes in patients with TMD and BMS were reported following MRI scanning. For each paper, the task differed, and these are displayed in Table 1. The areas of the brain that reported changes during task states for TMD and BMS are shown in Figure 3.

The areas of the brain consistently reported to have changes in task states for both TMD and BMS were the thalamus (Thal), secondary somatosensory area (S2), cingulate cortex (CC), insula (INS), basal ganglia (BG), and medial temporal lobe (mTL). The areas that had no reported changes during task states for TMD or BMS were the primary motor area (M1) and the supplementary motor area (SMA). In addition to this, BMS patients also had no reported change in the primary somatosensory cortex (S1).

### 3.7. Shared Brain Area Changes Irrespective of Task or Resting State

**Figure 4:**
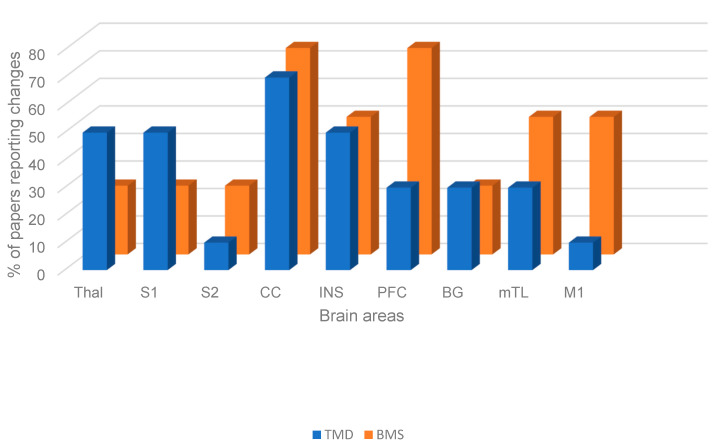
Figure showing shared brain changes in TMD and BMS irrespective of task or resting state.

**Figure 5:**
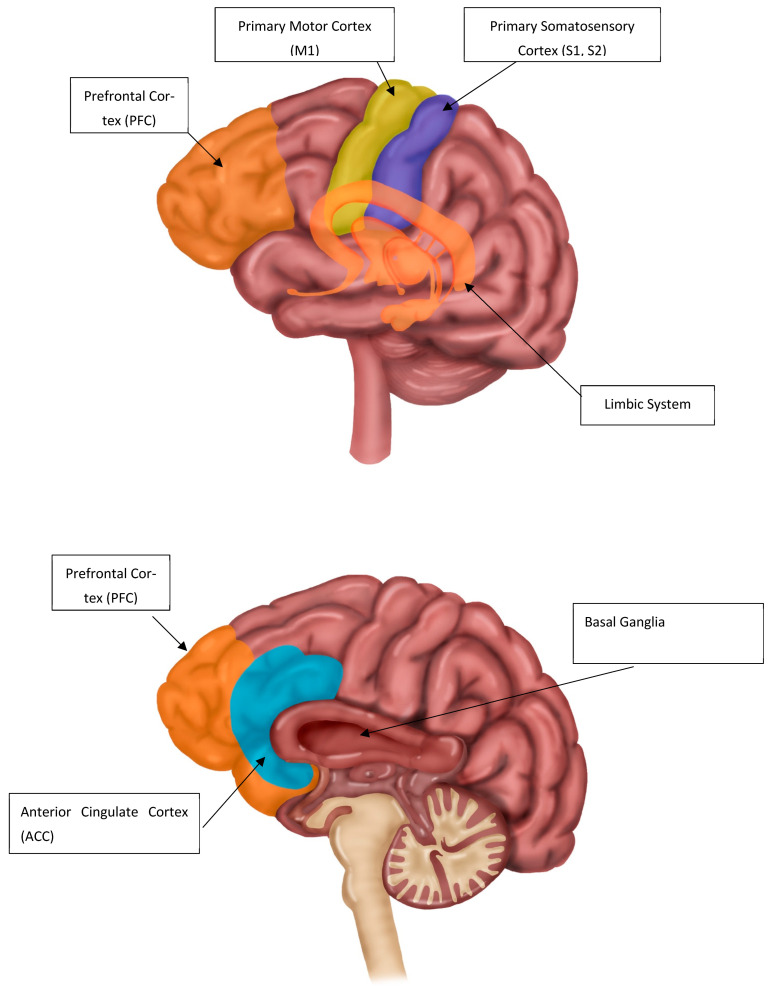
Illustrations of the brain areas highlighted in TMD and BMS.

Both resting-state and task-state changes were seen in multiple common brain areas; these included the thalamus, S1, S2, CC, insula, PFC, basal ganglia, mTL, and M1. These are shown in Figure 4 and in the brain model illustrated in Figure 5.

## 4. Discussion

This systematic review provides a synthesis of functional neuroimaging findings across two key subtypes of chronic orofacial pain (COFP): temporomandibular disorder (TMD) and burning mouth syndrome (BMS). Despite historical differences in classification and management [2,3,4,5,6], our findings demonstrate consistent alterations in the brain regions involved in both sensory-discriminative and affective-emotional components of pain processing. Specifically, shared changes were observed in the thalamus, somatosensory cortices (S1, S2), insula, cingulate cortex, prefrontal cortex, medial temporal lobe, basal ganglia, and primary motor cortex.

These findings support the ICD-11 classification of chronic primary orofacial pain [7], which conceptualises chronic pain not merely as a symptom but as a disease entity with shared central mechanisms and significant biopsychosocial impact. Notably, despite being anatomically distinct, TMD and BMS demonstrate convergent neural changes, suggesting a shared neurobiological underpinning consistent with central sensitisation and altered pain modulation [29]. This aligns with the growing body of literature highlighting the role of dysfunctional central processing in persistent orofacial pain conditions [30,31].

### 4.1. The Cingulate Cortex (CC) and Insula

The CC and insula areas of the brain are both known to be activated by painful stimuli. In addition to encoding pain intensity, they are involved in pain relief and coping, as well as the anticipation of and emotional processing of pain. They also have connections to other areas of the brain that have pain-processing functions, such as the PFC. It is suggested that these areas are part of an integration network, and dysfunction in these areas is implicated in the rumination and self-sustenance aspect of chronic pain [11,13,30,32].

Because of its role in the emotional and motivational aspects of pain, the CC can also be activated by strong emotional stimuli, and vice versa, by perpetuating the emotional response to pain [11]. There are two suggested reasons for the changes that are commonly seen in these areas of the brain in patients with COFP:

1. The CNS reduces the pain sensations felt by the patient by altering attention to pain and behavioural adjustment [10,11].
2. Increased stimulation of this area leads to changes that play a role in the maintenance of pain and the experience of normal oral sensations as pain [11,17].

### 4.2. The Prefrontal Cortex (PFC)

The PFC was one of the most consistently reported areas with neurological changes in patients with both TMD and BMS in this review. The PFC has roles in coping, modulating, and memory and emotional processing of pain. Behavioural responses to pain are directed by the PFC, and consistent changes seen in this area in COFP patients highlight the psychosocial aspect of this disorder [16,26]. These changes can manifest functionally as poor memory and cognitive impairment due to increased attention to on-going pain and mental health decline. These are all in agreement with the ICD-11 definition of chronic pain, which indicates that chronic pain causes “significant emotional distress and/or functional disability” [7].

### 4.3. The Limbic System

The thalamus was one of the most highlighted areas that experienced changes in patients with COFP. The thalamus is part of the limbic system; it receives peripheral pain information and projects this to the S1, as part of the thalamocortical pathway. The normal functions of the thalamus include affective and emotional aspects of pain, motivational learning, and generation and maintenance of pain [11,24]. The thalamus is also implicated when there is psychological distress in the form of depression and anxiety [24].

Dysfunction in this area can lead to the transition of acute to chronic pain, reduced inhibition of pain, and the inability to discriminate between normal sensations and pain, which, in turn, could lead to overloading of the CNS, affecting its ability to function normally [2,3,4,5,6,7,8,9,10,11,12,13,14,15,16,17,18,19,20,21,22,23,24].

Again, there are two arguments about the cause of the changes seen in the thalamus in patients with chronic pain [24]:

1. Changes may be due to compensatory analgesic adaptations that attempt to reduce the sensation of pain.
2. Changes cause an increased transmission of painful signals due to increased or decreased neural connections.

### 4.4. Motor System Involvement

In addition to their primary motor functions, the SMA and M1 have roles in planning, processing, memory, and attention to pain. This is especially true if pain is perceived as uncontrollable, which may not always be the case in chronic pain [24]. What was interesting in the results of this review was that these areas were only highlighted to have changes in resting-state tasks, even when the task required a motor function, such as clenching. There is evidence to show that patients with COFP alter their jaw movements to attempt to reduce the pain they feel. This may be due to abnormalities in descending tracts, which lead to abnormal muscle and motor activations, e.g., hyperactivity of masticatory muscles, distorted proprioception, and abnormal sensation. This, in turn, may cause parafunctional habits and chronic pain [19,30].

### 4.5. Future Research and Limitations of This Review

Nonetheless, this review has notable limitations. The studies varied in imaging protocols and patient inclusion criteria, and few accounted for potential confounders, such as medication use or psychological comorbidities. Most participants were female, reflecting epidemiological trends [8,9] but limiting generalisability. Future research should explore whether gender differences exist in relation to neural changes in COFP. Moreover, while this review’s search terms included several imaging techniques, the finally included articles focused only on structural and functional MRI. Other imaging modalities, such as PET and SPECT, warrant further exploration as they can provide valuable additional information related to the underlying molecular functions in the brain. Future studies should include these techniques to explore neuroimaging changes in COFP. Because of the heterogeneity in the measurement of neural changes, a meta-analysis could not be undertaken, and this further limited our ability to pool the results. Finally, we did not identify the grey literature or unpublished studies in this area, which may have led to some data being missed. That said, we contacted the authors of the included studies for missing or unclear data to ensure rigour in our data extraction process.

Future research should aim to standardise imaging methodology, adopt causal inference approaches to account for confounding [28], and investigate these conditions longitudinally. Such work could strengthen the evidence base for classifying COFP subtypes under a shared diagnostic framework and guide the development of targeted treatment pathways.

### 4.6. Clinical Implications

From a clinical perspective, these results reinforce the need for an integrated, interdisciplinary management approach. The current tendency to address TMD and BMS in isolation—both in clinical practice and research—may limit therapeutic effectiveness. Instead, recognising shared mechanisms may allow for cross-applicable, mechanism-based interventions, such as cognitive behavioural therapy, neuromodulation, or interdisciplinary pain rehabilitation programs. The results support the shift away from pharmacological and invasive interventions in favour of interdisciplinary, mechanism-informed management approaches, as recommended by the National Institute for Clinical Excellence and National Academies of Medicine for managing chronic primary pain and TMD, respectively [33,34]. Biobehavioural interventions targeting central mechanisms may offer improved outcomes and reduce treatment burden and have been shown to reduce long-term pain intensity and depression in chronic TMD sufferers [35]. We have recently shown that such approaches, if delivered as first-line treatments, result in cost savings by reducing consultation rates and avoiding invasive and irreversible treatments [35]. They can also be remotely delivered, which enhances accessibility and environmental sustainability [36].

## 5. Conclusions

This systematic review highlights consistent functional neuroimaging changes across the subtypes of chronic orofacial pain, specifically temporomandibular disorder and burning mouth syndrome. The convergence of alterations in key brain regions involved in pain processing and emotional regulation supports the ICD-11 classification [7] of chronic primary orofacial pain and suggests a shared central mechanism underlying these conditions. These findings underscore the need to move beyond a purely anatomical or symptom-based approach to diagnosis and management, advocating instead for a biopsychosocial, mechanism-based model of care. Recognition of shared neurobiological pathways may facilitate more effective, interdisciplinary treatment strategies and inform the development of unified clinical guidelines [33,34]. Further high-quality, standardised neuroimaging studies are needed to strengthen these findings and support translation into clinical practice.

## Figures and Tables

**Table 1:** Patient demographics included in this review.

Patient						Control						
Source	Diagnosis	Imaging Modality	Analysis Method	Resting/Task	Female	Male	Age (Years)	Assessment	Duration of Pain	Female	Male	Age
***Kucyi 2014*** ***[15]***	TMD	MRI (BOLD T2	FC	Resting	17		33.1 ± 11.9	NPS, PCS	minimum 3 months	17		32.2 ± 10.2
***Youssef 2014*** ***[16]***	TMD	MRI (STAR labelled T1)	CBF	Resting	12	3	44.9 ± 3.1	VAS, MPQ	11.4 ± 3.3 years	41	13	46.9 ± 2.1
***Younger 2010*** ***[17]***	TMD	MRI (FAST 3D-SPGR)	GMV	Resting	14		38 ± 13.7	NRS	4.4 ± 2.9 years	15		age-matched
***Ichesco 2012*** ***[18]***	TMD	MRI (T2spiral sequence)	FC	Resting	8		23–31	VAS, SF-MPQ, BPI, STPI	minimum 3 months	8		22–27
***Moayedi 2012*** ***[19]***	TMD	MRI (T1, DTI)	FA	Resting	17		33.1 ± 11.9	NPS	9.8 ± 8.25 years	17		32.2 ± 10.1
***Gustin 2012*** ***[20]***	TMD	MRI (BOLD T1)	BA, CBF, FA	Innocuous brushing of lower lip, little finger, and thumb	13	4	44 ± 3	VAS, MPQ	10.7 ± 2 years	27	26	41 ± 2
***Zhao 2011*** ***[21]***	TMD	MRI (T2)	BA	Clenching	11	3	33.7 ± 13.2	VAS, SCL−90	Not reported	7	7	23.7 ± 0.9
***Ichesco 2012*** ***[18]***	TMD	MRI (T2 spiral sequence)	FC	Elicited pain	8		23–31	VAS, SF-MPQ, BPI, STPI	minimum 3 months	8		22–27
***Gustin 2011*** ***[22]***	TMD	fMRI (QASL, DTI)	GMV, metabolite levels	Innocuous lip brushing	16	4	45.7 ± 2.9	VAS, MPQ, BDI, STATE	11.4 ± 3.3 years	25	6	46.8 ± 3.3
***Weissman-Fogel 2011*** ***[23]***	TMD	MRI (T1 (structural), T2 (functional))	Peak activation coordinates	nSTROOP, ncSTROOP, ecSTROOP	17		35.2 ± 11.6	NPS	9.3 ± 8.3 years	17		34 ± 9.9
***Sinding 2016*** ***[24]***	BMS	MRI (T1 3d IR/GR sequence)	GMC	Resting	7	5	59.4 ± 12.1	unanchored, unmarked scale	minimum 3 months	10	3	59 ± 3.4
***Khan 2014*** ***[25]***	BMS	MRI	GMV, FA, DTI, FC	Resting	9		54 ± 7.7	STAI	4 ± 4.8 years	9		age matched
***Albuquerque 2006*** ***[26]***	BMS	MRI (EPI sequence)	BA	Thermal stimulation	8		49.1 ± 10.1	SCL-90R, BDI, STAI	60.5 ± 38.4 months	8		50 ± 12.3
***Kohashi 2020*** ***[27]***	BMS	MRI (T2)	BA	Thermal stimulation	15		52.6 ± 6.3	Not reported	not reported	15		49 ± 8.4

FC: functional connectivity; CBF: cerebral blood flow, GMV: grey matter volume; FA: fractional anisotropy; BA: brain activation; GMC: grey matter concentration; DTI: Diffusion Tensor Imaging; WMH: white matter hyperintensities.

## Data Availability

The data that support the findings of this study were synthesised from previously published studies, which are publicly available. Data related to synthesis of the findings can be requested by contacting the corresponding author (VRA).

## Supplementary Materials

The following supporting information can be downloaded at https://www.mdpi.com/article/10.3390/dj13080340/s1: Supplementary File S1: Search Strategy for OVID Search.
