# Supplementary material for: Neuroimaging Signatures of Temporomandibular Disorder and Burning Mouth Syndrome: A Systematic Review
Source: Dent J (Basel). 2025 Jul 24;13(8):340. doi: 10.3390/dj13080340 (PMC12385566; doi:10.3390/dj13080340)
Supplement: Supplementary file 1 [file dentistry-13-00340-s001.zip › dentistry-3752813-supplementary.pdf]

Supplemental File I: Search Strategy for OVID Search

1. CRANIOMANDIBULAR DISORDERS/
2. (temporomandibular$ or temporo-mandibular).mp.
3. tmj.mp. or tmd.ti,ab.
4. exp MYOFASCIAL PAIN SYNDROMES/
5. (myofascial and (pain$ or disorder$ or dysfunction$)).mp.
6. (myofacial and (pain$ or disorder$ or dysfunction$)).mp.
7. exp Facial Pain/
8. (atypical and facial pain).mp.
9. (atypical and facial neuralgia).mp.
10. BURNING MOUTH SYNDROME/
11. (mouth and (pain$ or burning)).mp.
12. exp orofacial pain/
13. (atypical and orofacial pain).mp.
14. (atypical and odontol$).mp.
15. (atypical and toothache$).mp.
16. (atypical and tooth pain).mp.
17. phantom tooth pain.mp.
18. (persistent and tooth pain).mp.
19. (persistent and toothache$).mp.
20. PIOP.mp.
21. (persistant and orofacial pain).mp.
22. oral dysthesia/
23. or/1-22
24. exp brain imaging/
25. (CT or computed tomography).mp.
26. (MRI or magnetic resonance imaging).mp.
27. (positron emission tomography or PET).mp.
28. (functional magnetic resonance imaging or fMRI).mp.
29. (Single photon emission tomography or SPECT).mp.
30. (electroencephalogram or EEG).mp.
31. or/24-30
32. 23 and 31
33. quantitative.mp. [mp=ti, ab, hw, tn, ot, dm, mf, dv, kw, fx, dq, nm, kf, ox, px, rx, ui, sy]
34. 32 and 33
35. limit 34 to english language
36. limit 35 to full text
37. limit 36 to humans

Search Strategy for SCOPUS Search

TITLE-ABS-KEY ((*craniomandibular* AND *disorders*) OR (*temporomandibular* AND *disorders*) OR (*tmj*) OR (*myofascial* AND *pain* AND *syndromes*) OR (*myofascial* AND (*pain* OR *disorder* OR *dysfunction*)) OR (*myofacial* AND (*pain* OR *disorder* OR *dysfunction*)) OR (*facial* AND *pain*) OR (*atypical* AND *facial* AND *pain*) OR (*atypical* AND *facial* AND *neuralgia*) OR (*burning* AND *mouth* AND *syndrome*) OR (*mouth* AND (*pain* OR *burning*)) OR (*orofacial* AND *pain*) OR (*atypical* AND *orofacial* AND *pain*) OR (*atypical* AND *odontol*) OR (*atypical* AND *toothache*) OR (*atypical* AND *tooth* AND *pain*) OR (*phantom* AND *tooth* AND *pain*) OR (*persistent* AND *tooth* AND *pain*) OR (*persistent* AND *toothache*) OR (*piop*) OR (*persistent* AND *orofacial* AND *pain*) OR (*oral* AND *dysthesia*)) AND ((*brain* AND *imaging*) OR (*ct* OR *computed* AND *tomography*) OR (*mri* OR *magnetic* AND *resonance* AND *imaging*) OR (*positron* AND *emission* AND *tomography* OR *pet*) OR (*single* AND *photon* AND *emission* AND *tomography* OR *spect*) OR (*functional* AND *magnetic* AND *resonance* AND *imaging* OR *fmri*) OR (*electroencephalogram* OR *eeg*) AND (*quantitative*) AND NOT (*acute*)) AND (LIMIT-TO (*ACCESSTYPE(OA)*)) AND (LIMIT-TO (LANGUAGE , *"English"*))
